# Reciprocal restriction fragment length polymorphism (RFLP) analysis reveals mitochondrial heteroplasmy in *Caenorhabditis briggsae* hybrids

**DOI:** 10.17912/micropub.biology.001306

**Published:** 2024-08-09

**Authors:** Kevin Helwick, Joseph Ross

**Affiliations:** 1 Department of Biology, California State University, Fresno

## Abstract

Although mitochondria are typically inherited maternally, exceptions exist. We previously demonstrated that within-species crosses of *Caenorhabditis briggsae* result in paternal mitochondrial transmission, and it would be useful to know whether hybrids have only paternal mitochondria (homoplasmy) or paternal and maternal mitochondria (heteroplasmy). We developed a reciprocal restriction fragment length polymorphism analysis to separately detect paternal and maternal mitochondrial DNA. Using new hybrid lines, this approach revealed that some hybrids are heteroplasmous and others have become homoplasmous for the paternal mitotype. These results motivate additional investigation of how paternal mitochondrial transmission is apparently facile in *C. briggsae* .

**Figure 1. Reciprocal RFLP analysis of C. briggsae lines:**
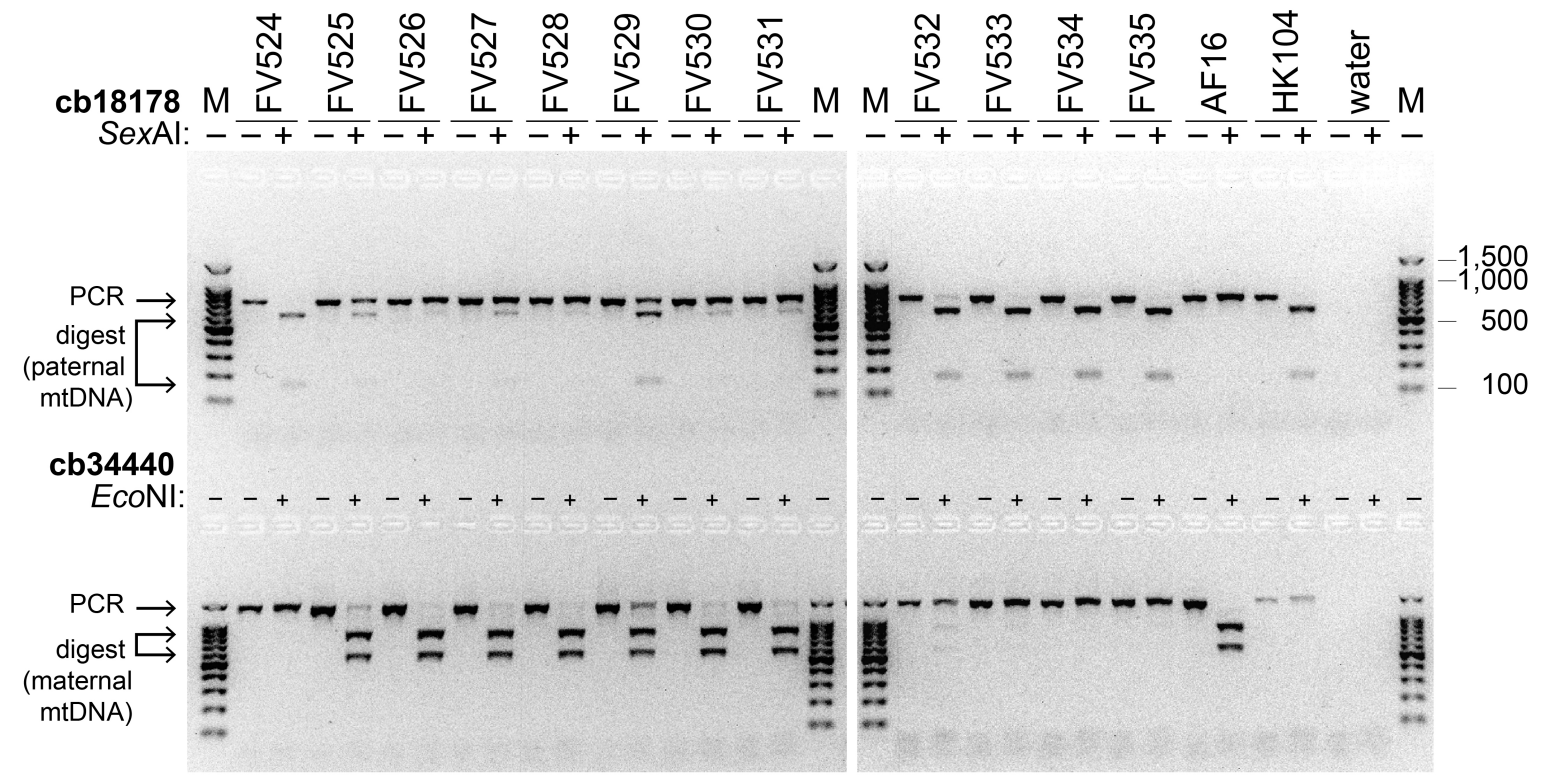
Each DNA sample ( HK104 x AF16 cybrid lines FV524 – FV535 , positive control parental strains AF16 and HK104 , and negative control water) was PCR amplified by the two RFLP primer pairs at left (top row cb18178; bottom row cb34440). After PCR, an aliquot of each amplicon was digested with the restriction enzyme shown at left ( *Sex* AI or *Eco* NI). The undigested and digested samples were loaded into adjacent wells (– indicates absence of restriction enzyme; + indicates presence of restriction enzyme). M: molecular weight ladder; some bands are labeled at right (bands are in 100 bp increments from 100–1,000 bp). Electrophoresis occurred on one agarose gel and the left and right halves were imaged separately; a single M lane in the middle was acquired in both images to allow accurate alignment of the images. The positive control templates establish the expected digest band sizes. For cb18178, both AF16 and HK104 mtDNA produce 800 bp mtDNA amplicons; only HK104 digests into 650+150 bp products. For cb34440, both AF16 and HK104 produce 1,445 bp mtDNA amplicons; only the AF16 amplicon digests into 865+580 bp products. Thus, for cb18178 RFLP analysis, presence of any digest band indicates the presence of paternal mtDNA amplicons. For cb34440 RFLP analysis, presence of any digest band indicates the presence of maternal mtDNA amplicons. When both RFLP assays for a line produce digest bands, this indicates the line is heteroplasmic.

## Description

Mitochondria are inherited maternally. However, paternal mitochondrial transmission (PMT) occurs, particularly in crosses of genetically diverse parents from the same or closely related species, e.g. (Chang et al., 2016; Fontaine et al., 2007; Gyllensten et al., 1991; Kvist et al., 2003; Lee & Willett, 2022; Ross et al., 2016; Sherengul et al., 2006; Shitara et al., 1998; Wolff et al., 2013) . These data suggest that cellular mechanisms regulating mitochondrial inheritance are not foolproof. Thus, any assumption that mitochondria are only maternally inherited is unwarranted and could lead to erroneous conclusions, for example in studies assuming that mitochondrial genotypes (mitotypes) represent maternal lineages in phylogenetics. A recent study found mitotype-dependent sex-specific differences in gene expression and lifespan (Li et al., 2024) , further underscoring the value of understanding how mitochondrial inheritance is regulated.

Various methods, including fluorescent labeling of paternal mitochondria and allele-specific polymerase chain reaction (PCR), have been used to track mitochondrial inheritance in various species, e.g. (Al Rawi et al., 2011; Lee & Willett, 2022; Sato & Sato, 2011) . We favor using restriction fragment length polymorphism (RFLP) analysis for detecting PMT. RFLP begins with PCR amplification of a region of the mitochondrial genome (mtDNA) using a pair of primers that anneals to both the maternal and paternal mitotypes. The PCR amplicon contains a single nucleotide polymorphism (SNP) in the paternal mtDNA that creates a restriction endonuclease site not present in the maternal amplicon. Restriction digest of the PCR amplicon is then used to evaluate whether an individual is homoplasmous maternal (only has maternal mitotypes: no digest products are observed on an agarose gel), or heteroplasmous (has both maternal and paternal mitotypes: both undigested and digested products are observed), or homoplasmous paternal (only has paternal mitotypes: all PCR amplicons digest).

Our previous work in *Caenorhabditis briggsae* , a close relative of *C. elegans* , employed crosses between the populations AF16 and HK104 . Serial backcrosses produced cytoplasmic-nuclear hybrids (cybrids) that should contain AF16 nuclear DNA (nDNA) and HK104 cytoplasm (including mtDNA). We developed an RFLP assay containing the SNP cb18178 (Koboldt et al., 2010) and demonstrated that HK104 amplicons digest while AF16 amplicons do not (Chang et al., 2016) . Thus, the cb18178 assay detects PMT from HK104 males into AF16 cytoplasm. Using this approach, we observed PMT by detecting paternal mitotypes in hybrid offspring (Chang et al., 2016; Ross et al., 2011, 2016) .

One drawback to the use of single RFLP assays is the ambiguous interpretation of undigested bands. Such bands either represent maternal amplicons lacking the restriction site or paternal amplicons that did not yet digest. Here, we introduce the approach of reciprocal RFLP to address this limitation. Two RFLP assays are used to investigate heteroplasmy: one assay digests only the maternal mtDNA amplicon, and a second digests only the paternal mtDNA amplicon.

First, we identified another SNP (cb34440) present in AF16 mtDNA that creates an *Eco* NI restriction site not present in HK104 mtDNA. The primers that amplify this SNP are GGGGCCTTAAAACAGTAAAAGG and CCTTTTGGGAGAAGTAAGATGC. They produce a 1,445 bp mtDNA amplicon from both AF16 and HK104 , but only the AF16 amplicon digests into 865+580 bp products ( Figure 1 ). We then created new cybrids to measure the frequency of PMT. Each line was initiated by mating a P0 generation HK104 male with a self-sperm depleted AF16 hermaphrodite. F1 hybrid hermaphrodites were self-sperm depleted and backcrossed to HK104 males. After four additional generations of HK104 backcrossing, each line was selfed by passaging three virgin hermaphrodites per generation for five generations to produce replicate lines FV524 – FV535 . Because the HK104 mitotype was only present in males, each line should be homoplasmous for the AF16 mitotype if strictly maternal mitochondrial inheritance occurs.

We purified DNA from the twelve lines and from AF16 and HK104 . These DNA and water were PCR amplified both with the cb34440 primers and the cb18178 primers in 20 µL reactions containing: 1x One *Taq* Master Mix (New England Biolabs), 0.5 µM each primer, and 10 ng template DNA. Half the volume of each cb18178 amplicon was digested as before (Chang et al., 2016) , and half the volume of each cb34440 amplicon was digested using the same protocol but with *Eco* NI (New England Biolabs). For each line, undigested and digested amplicons were electrophoresed in adjacent wells on a 2% agarose/1x TAE gel stained with SYBRSafe (ThermoFisher Scientific) and including 100 bp molecular weight ladder (Promega).

For cb18178 ( Figure 1, top row), the AF16 amplicon does not digest and the HK104 amplicon does, as expected (Chang et al., 2016) . Lack of digest product in AF16 suggests that this parental population is homoplasmic for the AF16 mitotype. Complete digestion of the HK104 amplicon suggests that this parental population is homoplasmic for the HK104 mitotype. All twelve experimental cybrid lines show cb18178 digest products ranging from faint (e.g. FV528 ) to pronounced (e.g. FV533 ), revealing pervasive PMT. Most of the experimental lines also have some undigested PCR product, raising the question whether these are maternal mtDNA amplicons (heteroplasmy) or paternal amplicons that have not yet digested (homoplasmy).

To distinguish between these outcomes, the cb34440 RFLP assay is informative. This assay digests the AF16 mtDNA amplicon and produces no digest of the HK104 amplicon ( Figure 1, bottom row). Some cybrid lines ( FV524 , FV533 , FV534 , FV535 ) produce no visible digest product, which suggests that they are homoplasmous for the paternal mitotype. The other eight cybrid lines produce digest products with both cb18178 and cb34440, which suggests that they are heteroplasmous. Thus, after five generations of paternal backcrossing and five generations of selfing, eight of twelve lines have inherited paternal mtDNA and are heteroplasmous. The other four lines contain no detectable maternal mtDNA and thus are homoplasmous for the paternal mitotype.

A key benefit of RFLP analysis is that it limits false positive results, which is essential when challenging the dogma that PMT does not occur. Exceptions are expected to be exceedingly rare. For example, *de novo* mutation of maternal mtDNA to create the same restriction site found in paternal mtDNA is possible but unlikely. Also, some species contain mtDNA sequences that have integrated into the nuclear genome (Bensasson et al., 2001) . These nuclear mitochondrial DNA sequences (NUMTs) will complicate evaluation of PCR-based mtDNA methods if mtDNA-specific primers also amplify NUMTs. The potential for generating false evidence of PMT by unknowingly PCR-amplifying NUMTs can be reduced when the maternal population used in a cross does not produce an RFLP digest product, which we show: AF16 produces no visible cb18178 digest ( Figure 1 ), suggesting that AF16 contains no cb18178 NUMTs.

A more critical concern is whether the paternal population ( HK104 ) has a NUMT containing the primer sites and the restriction site. In such a case, offspring could inherit this nuclear allele and at least some of the PCR amplicons would digest, giving the appearance of PMT. Figure 1 shows some cb18178 digest occurring in HK104 and in all twelve replicate cybrid lines. However, presence of a cb18178 NUMT in HK104 is unlikely. If the lines that appear homoplasmous for the paternal mitotype did contain a cb18178 NUMT, then those lines would still be expected to have maternal mitotypes. However, lines like FV524 produce no cb34440 digest product and thus contain no detectable maternal mitotypes and must contain paternal mitotypes.

We used reciprocal RFLP analysis to assess the heteroplasmy status of twelve new *C. briggsae* cybrid lines. We observed extensive heteroplasmy as well as loss of heteroplasmy for paternal homoplasmy. None of the twelve replicate lines has maternal homoplasmy, which is what should exist if strict maternal mitochondrial inheritance occurs. Future work in this system will continue to explore the temporal dynamics of PMT in within-species crosses, as well as how genetic divergence between parents might influence PMT frequency.

## Reagents

**Table:** 

**Strain**	**Genotype**	**Available From**
AF16	*C. briggsae* wild isolate	CGC*
HK104	*C. briggsae* wild isolate	CGC
FV524	*C. briggsae* HK104 x AF16 cybrid	Authors
FV525	*C. briggsae* HK104 x AF16 cybrid	Authors
FV526	*C. briggsae* HK104 x AF16 cybrid	Authors
FV527	*C. briggsae* HK104 x AF16 cybrid	Authors
FV528	*C. briggsae* HK104 x AF16 cybrid	Authors
FV529	*C. briggsae* HK104 x AF16 cybrid	Authors
FV530	*C. briggsae* HK104 x AF16 cybrid	Authors
FV531	*C. briggsae* HK104 x AF16 cybrid	Authors
FV532	*C. briggsae* HK104 x AF16 cybrid	Authors
FV533	*C. briggsae* HK104 x AF16 cybrid	Authors
FV534	*C. briggsae* HK104 x AF16 cybrid	Authors
FV535	*C. briggsae* HK104 x AF16 cybrid	Authors

* *Caenorhabditis* Genetics Center
